# An Integrated Multi-Omics Analysis Identifies Oxeiptosis-Related Biomarkers in Diabetic Retinopathy

**DOI:** 10.3390/biomedicines13112789

**Published:** 2025-11-15

**Authors:** Jiaoyu Deng, Pengfei Ge, Ying Gao, Hong-Ying Li, Yifan Lin, Yangyang Lu, Haiyue Xie, Dianbo Xu, Ping Xie, Zizhong Hu

**Affiliations:** 1 Department of Ophthalmology, The Affiliated Jiangning Hospital of Nanjing Medical University, Nanjing 211000, China; dengjiaoyu@jnyy10.wecom.work; 2 Department of Ophthalmology, The First Affiliated Hospital of Nanjing Medical University, Nanjing 210029, China; 3 Department of Ophthalmology, Sir Run Run Hospital, Nanjing Medical University, Nanjing 211112, China; 4 Department of Gynecology, The Affiliated Jiangning Hospital of Nanjing Medical University, Nanjing 211000, China

**Keywords:** diabetic retinopathy, oxeiptosis, CASP2, PLEC, biomarkers, mendelian randomization, machine learning

## Abstract

**Background:** Diabetic retinopathy (DR), a leading cause of blindness, lacks early biomarkers and mechanism-targeted therapies. While oxidative stress drives DR pathogenesis, the role of oxeiptosis—a reactive oxygen species-induced, caspase-independent cell death pathway—remains largely unexplored. **Methods:** We integrated transcriptomic profiling (GSE221521: 69 DR vs. 50 controls), two-sample Mendelian randomization (MR) using blood cis-eQTLs (GTEx) as instruments and DR GWAS (FinnGen R12) as outcome, machine learning-based feature selection (SVM-RFE and Boruta algorithms), and single-cell RNA sequencing (scRNA-seq) analysis (GSE165784). Functional enrichment, immune deconvolution (CIBERSORT), and diagnostic nomogram construction were performed. We validated the key genes using human retinal microvascular endothelial cells (hRMECs) treated with high glucose (30 mM). **Results:** Oxeiptosis scores were elevated in DR blood samples (*p* < 0.001). MR analysis identified five putative causal genes: CASP2 (OR = 1.067), PLEC (OR = 1.035) and FBN2 (OR = 1.016) as risk factors, and CYP27A1 (OR = 0.960) and GPD2 (OR = 0.958) as protective factors. SVM-RFE and Boruta algorithms confirmed CASP2 and PLEC as hub genes. A nomogram incorporating both genes achieved robust DR prediction (AUC = 0.811). Functional analysis associated these genes with innate immune activation and extracellular matrix reorganization. Single-cell transcriptomics revealed PLEC was markedly overexpressed in disease-relevant cells (fibroblasts, endothelial cells), whereas CASP2 exhibited a distinct pattern, with notable enrichment in retinal CD8+ T cells. Both genes were associated with a pro-inflammatory shift in the immune landscape. Their upregulation was validated in independent datasets and high-glucose-stressed retinal cells. **Conclusions:** This study establishes an integrated multi-omics framework implicating oxeiptosis-related pathways in DR and nominates CASP2 and PLEC as putatively causal, biologically relevant candidate biomarkers and potential therapeutic targets.

## 1. Introduction

Diabetic retinopathy (DR) stands as a particularly severe microvascular complication of diabetes. Its global prevalence, exceeding 103 million cases, is coupled with a profound public health impact as the foremost cause of preventable blindness in working-aged adults [1]. With the global diabetes epidemic projected to reach 700 million cases by 2045, DR prevalence is expected to increase to approximately 160 million cases, creating an unprecedented burden on healthcare systems globally [1,2]. Despite significant therapeutic advances including anti-VEGF therapies, laser photocoagulation, and surgical interventions, current DR management remains largely reactive, targeting late-stage complications rather than addressing underlying pathogenic mechanisms [3,4]. This approach results in suboptimal outcomes and limited prevention strategies, highlighting the urgent need for novel mechanistic insights and predictive biomarkers for early intervention.

Traditional DR research has predominantly focused on established pathways including hyperglycemia-induced oxidative stress, inflammation, and angiogenesis [5]. However, emerging evidence suggests that alternative cell death mechanisms may play crucial roles in retinal degeneration that extend beyond classical apoptotic pathways. Oxeiptosis, a recently discovered cell death modality first characterized in 2018, represents a caspase-independent pathway triggered by reactive oxygen species (ROS) and mediated through the KEAP1/PGAM5/AIFM1 signaling axis [6,7].

This pathway is mechanistically and phenotypically distinct from other ROS-mediated cell death pathways. The core cascade involves ROS-sensing by KEAP1, which then recruits PGAM5 to dephosphorylate AIFM1 at serine-116, leading to its nuclear translocation and caspase-independent cell death [8,9,10]. This mechanism clearly differentiates oxeiptosis from ferroptosis, an iron-dependent process driven by lipid peroxidation and GPX4 inhibition [11,12], and from pyroptosis, an inflammatory cell death executed by caspase-mediated gasdermin D cleavage [12,13]. Notably, oxeiptosis maintains an anti-inflammatory profile, contrasting sharply with the pro-inflammatory outcomes of pyroptosis and ferroptosis [6,7].

While the core oxeiptotic pathway is caspase-independent, its regulation and interplay with other cell death components remain incompletely understood. This complexity suggests that upstream regulators or parallel pathways could critically influence the oxeiptotic process and its pathological outcomes. Although initial studies have implicated oxeiptosis in pathogen clearance and oxidative stress responses, its role in diabetic complications, particularly DR, remains largely unexplored [14,15]. Our study therefore aims to investigate the contribution of this novel cell death pathway to DR pathogenesis.

Mendelian randomization (MR) overcomes confounding by leveraging genetic variants as instrumental variables for causal inference [16]. When integrated with single-cell RNA sequencing—which decodes retinal cellular diversity [17]—and machine learning for biomarker prioritization [18], this approach offers unprecedented power to identify therapeutically actionable targets. No study has yet applied this integrated framework to investigate oxeiptosis in DR, leaving three critical gaps: (1) putative causal links between oxeiptosis and DR development, (2) cell-type-specific pathway activation dynamics, and (3) diagnostic utility of oxeiptosis signatures. We therefore designed an oxeiptosis-guided integrative bioinformatics strategy utilizing peripheral blood transcriptomes as a discovery cohort to identify candidate genes. This comprehensive framework incorporated putative causal inference via MR, machine learning-based feature selection, and multi-level validation including single-cell transcriptomics and experimental models. This work aims to investigate the potential contribution of oxeiptosis-related pathways to DR pathogenesis and identify independent candidate biomarkers with clinical translational potential for early intervention.

## 2. Materials and Methods

### 2.1. Overall Analytical Workflow

**Figure 1:**
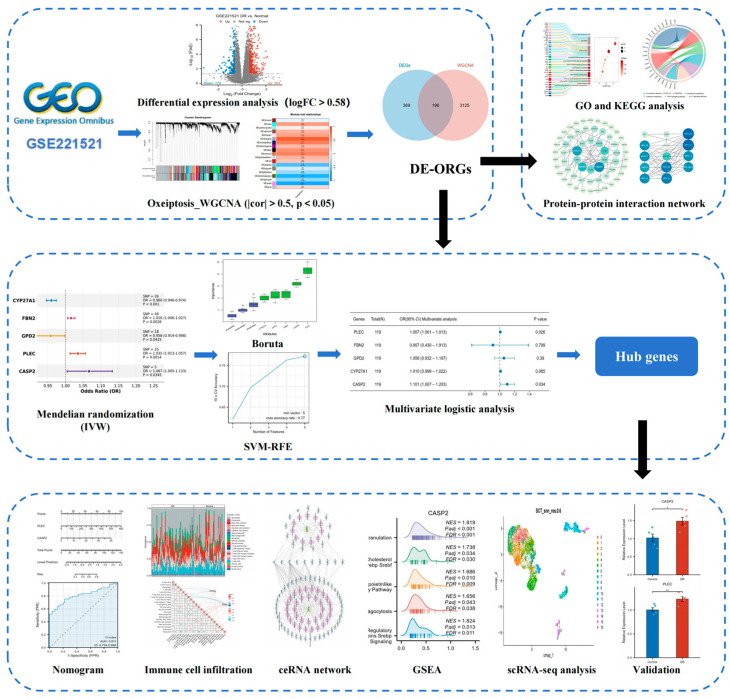
Study workflow.

The overall analytical pipeline comprised the following key steps: (1) Data Acquisition: Publicly available transcriptomic datasets from blood and fibrovascular membranes (FVM), along with single-cell RNA-seq data (scRNA-seq), were acquired as the foundational data layers. (2) Signature Screening in Discovery Cohort: The blood transcriptome discovery cohort (GSE221521) was analyzed to identify differentially expressed genes (DEGs) and to derive an oxeiptosis activity score, which guided a weighted gene co-expression network analysis (WGCNA). (3) Causal Inference: The intersection of DEGs and oxeiptosis-associated co-expression modules was defined as candidate genes, which were then subjected to Mendelian randomization (MR) analysis to infer putative causal relationships with DR. (4) Hub Gene Prioritization: Genes supported by MR evidence underwent a two-stage machine learning feature selection process to pinpoint the most robust hub genes, which were subsequently incorporated into a diagnostic nomogram. (5) Multi-level Validation: The expression and relevance of the identified hub genes were rigorously validated through multiple approaches: examining their expression in an independent retinal tissue dataset, functional characterization via gene set enrichment and immune landscape analysis, and mapping their cellular expression using single-cell transcriptomics. (6) Experimental Confirmation: The final step involved experimental validation of hub gene in a high-glucose cell model of DR. A visual summary of this pipeline is provided in Figure 1.

### 2.2. Data Acquisition

Transcriptomic data for DR were obtained from two Gene Expression Omnibus (GEO; https://www.ncbi.nlm.nih.gov/geo/) (accessed on 1 August 2025) datasets. The discovery cohort was derived from the GSE221521 dataset (Platform GPL24676) [19], which comprises high-throughput sequencing data from 119 blood specimens (69 DR vs. 50 control). Peripheral blood was chosen for its clinical accessibility and to capture the systemic inflammatory state inherent to DR. For validation of gene expression in the primary target tissue, we employed the GSE60436 dataset (Platform GPL6884) [20], containing microarray data from 9 FVM samples (6 DR and 3 control). The raw data were normalized using the limma package in R [21], including quantile normalization and log_2_ transformation. Additionally, we incorporated a scRNA-seq dataset (GSE165784 [22]) generated from FVM tissues of 5 proliferative DR patients.

Nineteen oxeiptosis-related genes (ORGs) were identified from GeneCards [23] (Table S1).

Blood cis-expression quantitative trait loci (cis-eQTL) data for individuals of European ancestry (EUR) were acquired from the Genotype-Tissue Expression (GTEx) consortium (version 8; https://gtexportal.org/home/) (accessed on 1 August 2025). Genome-wide association study (GWAS) summary statistics for DR (finngen_R12_DM_RETINOPATHY) were retrieved from the FinnGen R12 database (https://r12.finngen.fi) (accessed on 1 August 2025), encompassing 16,380,459 single-nucleotide polymorphisms (SNPs) across 216,666 individuals (14,584 DR cases and 202,082 controls).

### 2.3. Screening of Differentially Expressed ORGs (DE-ORGs) and Enrichment Analysis

We employed the DESeq2 package (version 1.42.1) [24] to identify DEGs between DR and control samples in the GSE221521 dataset, using thresholds of |log2fold change (FC)| > 0.58 and an adjusted *p*-value < 0.05.

Single-sample gene set enrichment analysis (ssGSEA) scores for the ORGs were computed using the GSVA package (version 1.50.5) [25] to compare DR and control groups (*p* < 0.05). These ORG scores were then employed as sample traits for WGCNA. Utilizing the WGCNA package (version 1.72-5) [26], modules of co-expressed genes correlating with sample traits were identified. Outliers were removed based on the Euclidean distance of sample expression profiles. A soft-thresholding power (β = 20) was selected to achieve scale-free topology (scale-free topology fitting index R^2^ = 0.8). Gene adjacency and topological overlap matrices were calculated, followed by hierarchical clustering with dynamic tree cutting (minimum module size = 20 genes). Modules exhibiting significant correlations with the traits (|correlation (cor)| > 0.5, *p* < 0.05) were classified as key modules.

DE-ORGs were derived from the intersection of the DEGs and genes within the key WGCNA modules. To elucidate the biological functions of the DE-ORGs, we analyzed their enrichment for Gene Ontology (GO) terms and Kyoto Encyclopedia of Genes and Genomes (KEGG) pathways using the “clusterProfiler” (v4.10.1) and “org.Hs.eg.db” (v3.18.0) packages [27]. Additionally, a protein–protein interaction (PPI) network was constructed using the STRING database [28] (https://cn.string-db.org/; minimum interaction confidence score > 0.5) (accessed on 1 August 2025) and visualized using Cytoscape (version 3.10.2).

### 2.4. Mendelian Randomization Analysis

We applied two-sample MR via the TwoSampleMR package (version 0.6.17) [29] to assess the potential causal relationships between DE-ORGs (exposure) and DR (outcome). Instrumental variables (IVs) were defined as cis-eQTLs for DE-ORGs from GTEx version 8 whole blood data (*p* < 5 × 10^−6^). Independent IVs were selected through linkage disequilibrium (LD) clumping (R^2^ < 0.01, clumping window = 100 kb) using PLINK (version 2.0) and 1000 Genomes European reference panel. Corresponding SNP effects for DR were extracted from GWAS summary statistics, with allele alignment performed to ensure strand consistency. Weak IVs (F-statistic < 10) were excluded. Causal estimates were generated using five methods implemented in TwoSampleMR: inverse-variance weighted (IVW) [30] as the primary approach (*p* < 0.05), supplemented by MR-Egger [31], and weighted mode [32], simple mode [29], and weighted median [33]. Odds ratios (ORs) derived from IVW indicated effect direction (OR > 1: risk factor; OR < 1: protective factor). Sensitivity analyses assessed heterogeneity using Cochran’s Q-test, evaluated horizontal pleiotropy via the MR-Egger intercept test and the global test of MR-PRESSO, and employed leave-one-out analysis to test the robustness of the findings. The discriminatory ability of the genes with putative causal evidence was further evaluated using receiver operating characteristic (ROC) curves generated by the pROC package (version 1.18.5), with the area under the curve (AUC) as the metric.

### 2.5. Hub Gene Identification and Nomogram Construction

Genes with putative causal evidence from the MR analysis underwent feature selection using two complementary methods: the Boruta algorithm (Boruta R package version 8.0.0) [34] for all-relevant feature identification (“Confirmed” genes), and support vector machine recursive feature elimination (SVM-RFE) implemented via “e1071” package (version 1.7-14) [35]. This dual strategy was employed to ensure that the final hub genes were not only biologically informative but also formed a minimal set with strong discriminatory power for building a robust diagnostic model. SVM-RFE utilized a linear kernel with 10-fold cross-validation, selecting optimal gene subsets through feature weight ranking. Genes consistently identified by both approaches were retained. Multivariable logistic regression identified hub genes independently associated with DR status (*p* < 0.05), reporting ORs with 95% confidence intervals (CIs). The expression patterns of hub genes were subsequently examined in the independent FVM dataset (GSE60436) to assess their relevance in the target pathological tissue. The “rms” package (version 6.7-1) was used to construct a diagnostic nomogram incorporating these hub genes. Model evaluation included ROC analysis, calibration plots assessing prediction–observation agreement, and decision curve analysis (DCA) quantifying clinical net benefit (“rmda” version 1.6) [36].

### 2.6. Gene Set Enrichment Analysis (GSEA) of Hub Genes

Samples from the GSE221521 dataset were dichotomized into high- and low-expression groups based on median expression levels of hub genes. DEGs between these groups were identified using “DESeq2” (version 1.42.1) [24]. GSEA was performed using the “clusterProfiler” package (version 4.10.1) [27] against the Molecular Signatures Database C2 canonical pathways collection (c2.cp.all.v2022.1.Hs.symbols.gmt). Significantly enriched pathways were defined by an adjusted *p*-value < 0.05 and false discovery rate (FDR) < 0.25.

### 2.7. Immune Infiltration Analysis

To quantify the immune cell composition, we applied the CIBERSORT deconvolution algorithm (version 0.1.0) [37] to the GSE221521 dataset. The analysis was performed using the standardized LM22 signature matrix and 100 permutations, with results filtered to include only samples achieving a CIBERSORT output of *p* < 0.05 for subsequent analyses. Differential immune cell enrichment between DR and control groups was assessed through comparative analysis (*p* < 0.05). Furthermore, to explore the associations between the candidate genes and the systemic immune landscape, the interrelationships between the expression levels of hub genes and the abundances of immune cell types were assessed using Spearman’s rank correlation analysis. 

### 2.8. scRNA-Seq Analysis

scRNA-seq data from five DR FVM samples were processed using “Seurat” (version 5.2.1) [38]. Quality control was performed by retaining genes detected in at least three cells and cells with a minimum of 200 detected genes. Additional filtering excluded cells with high mitochondrial gene expression (>20%), low complexity (log10GenesPerUMI < 0.8), or extreme RNA counts (<200 or >50,000). Doublets were identified and removed using “scDblFinder” (version 1.16.0) [39]. The SCTransform method was employed for data normalization, regressing out potential confounding effects from cell cycle scores (S.Score, G2M.Score, and CC.Difference) and mitochondrial ratio. Batch effects across samples were corrected using “Harmony” (version 1.2.3) [40]. Dimensionality reduction was performed via principal component analysis using variable features, with the top 25 principal components utilized for constructing a shared nearest neighbor graph and unsupervised clustering at a resolution of 0.6. Cell type annotation was conducted using ScType (version 1.0) [41] with tissue-specific marker databases for ocular tissues and immune system. The complete list of marker genes used for all cell types is provided in Table S2. Cell–cell communication networks were analyzed using “CellChat” (version 1.6.1) [42] to identify significant ligand-receptor interactions among different cell types. The expression profiles of hub genes across the annotated cell clusters were specifically visualized through feature plots and dot plots.

### 2.9. Competing Endogenous RNA (ceRNA) Network Construction

Experimentally validated microRNA (miRNA) interactions with hub genes were identified using the ENCORI platform (version 3.0; https://rnasysu.com/encori/) (accessed on 3 August 2025) with stringent filtering (≥5 supporting CLIP-seq datasets) [43]. Competing endogenous long non-coding RNAs (lncRNAs) targeting these miRNAs were subsequently screened. The integrated mRNA-miRNA-lncRNA ceRNA network was constructed and visualized in Cytoscape (version 3.10.2).

### 2.10. Cell Culture and RT-qPCR Validation

Human Retinal Microvascular Endothelial Cells (hRMECs, Jennio Biotech, Guangzhou, China, JNO-H0622) were maintained at 37 °C with 5% CO_2_ in endothelial cell growth medium containing 10% fetal bovine serum (FBS) and 1% penicillin-streptomycin. A DR cellular model was established by exposing cells to high glucose (HG, 30 mM D-glucose) for 48 h, with normal glucose (NG, 5.5mM D-glucose) as control.

Extraction of total RNA was carried out with an RNA rapid purification kit (Beyotime, Cat# R0026). cDNA synthesis was performed with the PrimeScript RT reagent kit (Takara, Cat# RR036A), followed by quantitative real-time PCR (RT-qPCR) using ChamQ Blue Universal SYBR qPCR Master Mix(Vazyme, Nanjing, China, Cat# Q312) on a 7500 Fast Real-Time Pcr System.

Gene-specific primer sequences were: GAPDH primers: forward GTCTTCACTACCATGGAGAAGG, reverse TCATGGATGACCTTGGCCAG; CASP2 primers: forward TGGTTCTTCAGTACCTCTACCAG, reverse GTTGGCCTTACAGCGGTAGAT. PLEC primers: forward CGATGCGACAACTTCACCTC, reverse GCCGGTACACCTTGTTCATGT. Relative gene expression was calculated by the 2^−ΔΔCt^ method normalized to GAPDH endogenous control. Data represent three independent biological replicates.

### 2.11. Statistical Analysis

All statistical computations were conducted using R (version 4.3.3). Data distribution normality was assessed via the Shapiro–Wilk test, with parametric tests applied when *p* ≥ 0.05. Group comparisons utilized unpaired two-sample Student’s *t*-test (normally distributed data) or Mann–Whitney U test (non-normally distributed data). A two-tailed *p* < 0.05 defined statistical significance.

## 3. Results

### 3.1. Screening of DEGs and Key Module Genes

**Figure 2:**
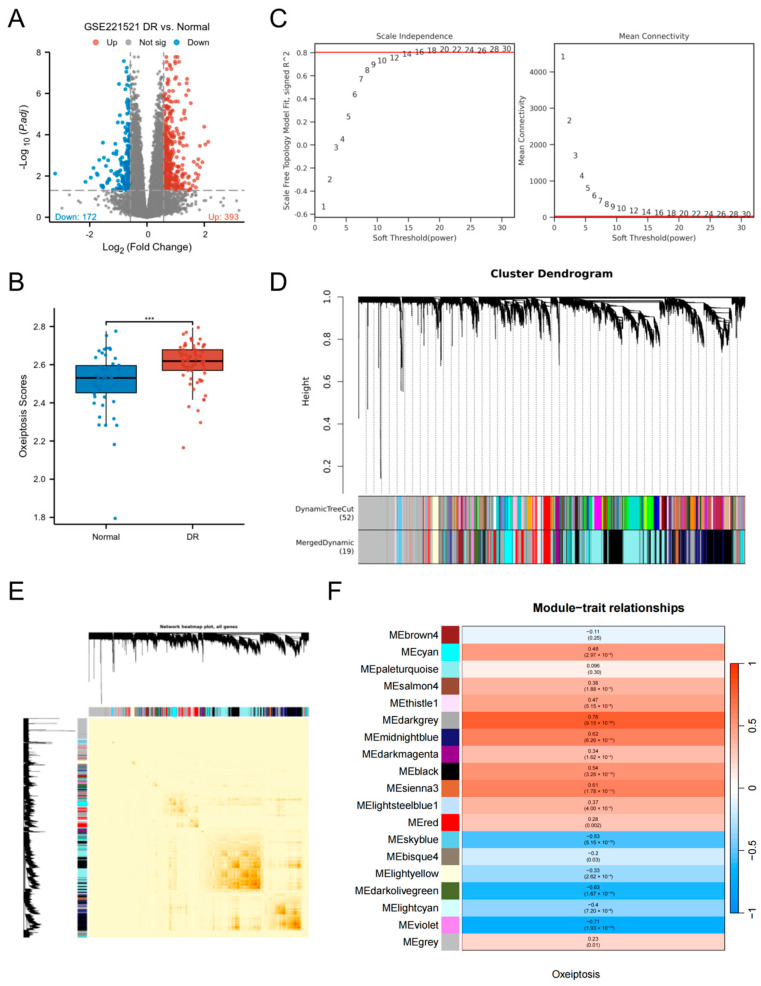
Screening of DEGs and key module genes. (**A**) Volcano plot of DEGs between DR and control groups in the GSE221521 dataset. (**B**) Comparison of oxeiptosis scores between the DR and control groups. (**C**) Analysis of network topology for various soft-thresholding powers (β) to determine the scale-free fit index. The optimal soft-thresholding power was determined to be β = 20. (**D**) Hierarchical clustering dendrogram of co-expressed genes, with different colors representing distinct modules. (**E**) Heatmap of the topological overlap matrix (TOM) depicting correlations among all module genes. Light color represents low overlap and darker red represents higher overlap. (**F**) Heatmap of module-trait relationships (Pearson’s correlation) between module eigengenes and the oxeiptosis score. (*** *p* < 0.001).

Analysis of the GSE221521 dataset identified 565 DEGs between the DR group and controls, with 393 upregulated and 172 downregulated (Figure 2A). Using the ssGSEA algorithm to score samples based on ORGs, we observed significantly elevated oxeiptosis scores in the DR group (*p* < 0.001; Figure 2B). For WGCNA analysis, genes with low expression variation (standard deviation ≤ 0.5) were filtered, retaining 7790 genes across 119 samples. A soft-threshold power (β = 20) achieved scale-free network topology (Figure 2C). Hierarchical clustering with dynamic tree cutting delineated 19 co-expression modules, visualized in a topological overlap matrix (TOM) heatmap (Figure 2D,E). The grey module, representing unassigned genes, was excluded from downstream analysis. Module-trait correlation analysis using Pearson’s method revealed 7 non-grey modules significantly associated with oxeiptosis score (|cor| > 0.5, *p* < 0.05; Figure 2F), comprising 3321 genes: darkgrey (n = 784), midnightblue (n = 515), black (n = 1536), sienna3 (n = 81), skyblue (n = 106), darkolivegreen (n = 214), and violet (n = 85).

### 3.2. Functional Enrichment and PPI Network of DE-ORGs

**Figure 3:**
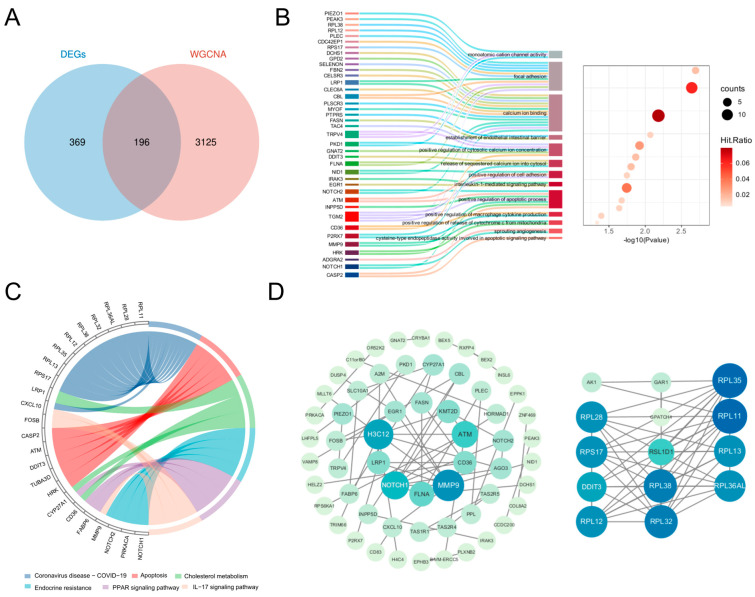
Identification of DE-ORGs and PPI network. (**A**) Venn diagram showing the overlap of DEGs and key module genes from WGCNA, with the intersection defined as DE-ORGs. (**B**) GO enrichment analysis of DE-ORGs, showing significantly enriched biological processes. (**C**) KEGG enrichment result for DE-ORGs. (**D**) PPI network of the DE-ORGs constructed using the STRING database.

Intersection analysis of DEGs and key WGCNA module genes identified 196 DE-ORGs (Figure 3A; Table S3). Functional enrichment analysis revealed significant associations with critical biological processes. GO enrichment (Figure 3B) demonstrated involvement in three functional clusters: apoptosis regulation (including positive regulation of cytochrome c release and caspase activity), inflammatory signaling (encompassing interleukin-1-mediated signaling and macrophage cytokine production), and ion homeostasis (notably calcium ion binding and cation channel activity), with complete term listings available in Table S4. KEGG analysis further confirmed enrichment in five key pathways: Apoptosis, IL-17 signaling, PPAR signaling, Endocrine resistance, and Cholesterol metabolism (Figure 3C). A comprehensive PPI network of these DE-ORGs was subsequently constructed to elucidate potential functional relationships (Figure 3D).

### 3.3. Hub Gene Screening

**Figure 4:**
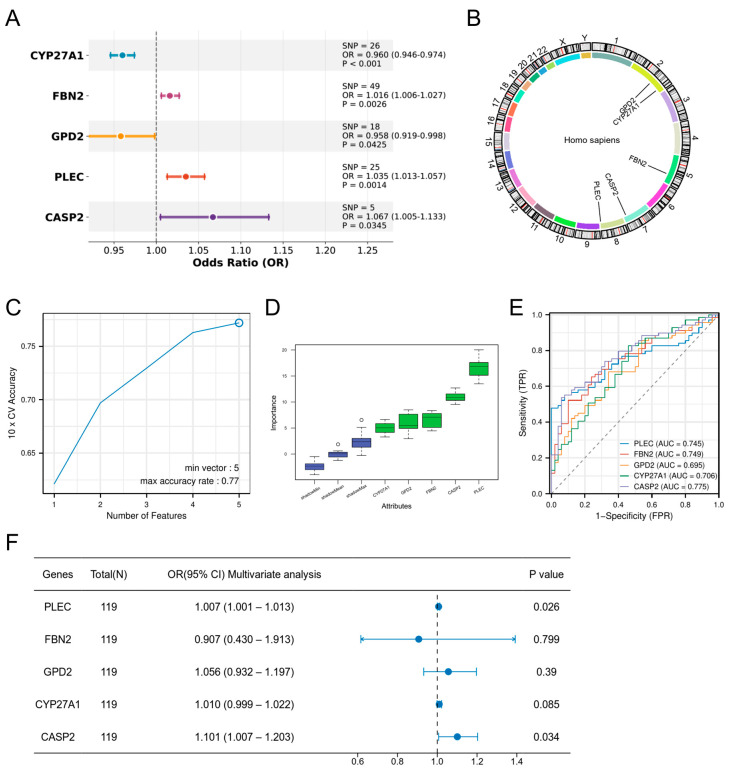
Screening of hub genes. (**A**) MR analysis identifying five DE-ORGs with potential causal effects on DR. ORs and 95% CIs are derived from the IVW method. (**B**) Chromosomal locations of the five candidate causal DE-ORGs. (**C**) Feature selection for the five candidate genes using the SVM-RFE algorithm. (**D**) Feature importance of the five candidate genes assessed by the Boruta algorithm (**E**) ROC curves demonstrating the diagnostic potential of the five candidate genes for DR. (**F**) Multivariable logistic regression analysis confirming CASP2 and PLEC as independent risk factors for DR.

MR analysis using 196 DE-ORGs as exposure factors and DR as outcome identified five genes with putative causal evidence via IVW estimation: CYP27A1 and GPD2 as protective factors (OR < 1, *p* < 0.05), and FBN2, PLEC, and CASP2 as risk factors (OR > 1, *p* < 0.05) (Figure 4A). Scatter plots demonstrated positive exposure-outcome correlations for CASP2, CYP27A1, FBN2, and PLEC, whereas GPD2 showed negative correlation (Figure S1). Forest plots quantified positive effect sizes for PLEC, CASP2, and FBN2, contrasting with negative effects for CYP27A1 and GPD2 (Figure S2). Funnel plots indicated symmetric distribution of instrumental variables (Figure S3). Sensitivity analyses confirmed absence of heterogeneity (*p* > 0.05, Table S5) and horizontal pleiotropy (*p* > 0.05, Table S6), while leave-one-out analysis validated result robustness without influential outliers (Figure S4). Chromosomal locations are mapped in Figure 4B.

To rigorously validate the strength of the genetic instruments and mitigate concerns of weak instrument bias, we calculated the F-statistics for all instrumental variables used in the MR analysis. The results demonstrated that all genetic instruments were strong. The mean F-statistics (and ranges) for each gene were as follows: CASP2: 51.10 (40.89–66.41); PLEC: 152.81 (20.55–518.86); FBN2: 167.67 (20.75–325.28); CYP27A1: 121.59 (109.03–135.82); GPD2: 41.42 (20.84–62.82). All values, including the minimum for each gene, were substantially above the standard threshold of 10, confirming the robustness of our causal inference (See Supplementary Table S7 for detailed values per SNP).

Subsequent validation via SVM-RFE (Figure 4C) and Boruta algorithm (Figure 4D) corroborated the functional importance of all five genes. The diagnostic capacity of the five candidate genes was evaluated by ROC analysis (Figure 4E), with AUCs as follows: CASP2 (0.775), PLEC (0.745), FBN2 (0.749), CYP27A1 (0.706), and GPD2 (0.695). Multivariable logistic regression confirmed CASP2 and PLEC as independent risk factors (CASP2: OR = 1.101, 95% CI 1.007–1.203, *p* = 0.034; PLEC: OR = 1.007, 95% CI 1.001–1.013, *p* = 0.026), establishing them as hub genes (Figure 4F).

### 3.4. Nomogram Construction Based on Hub Genes

**Figure 5:**
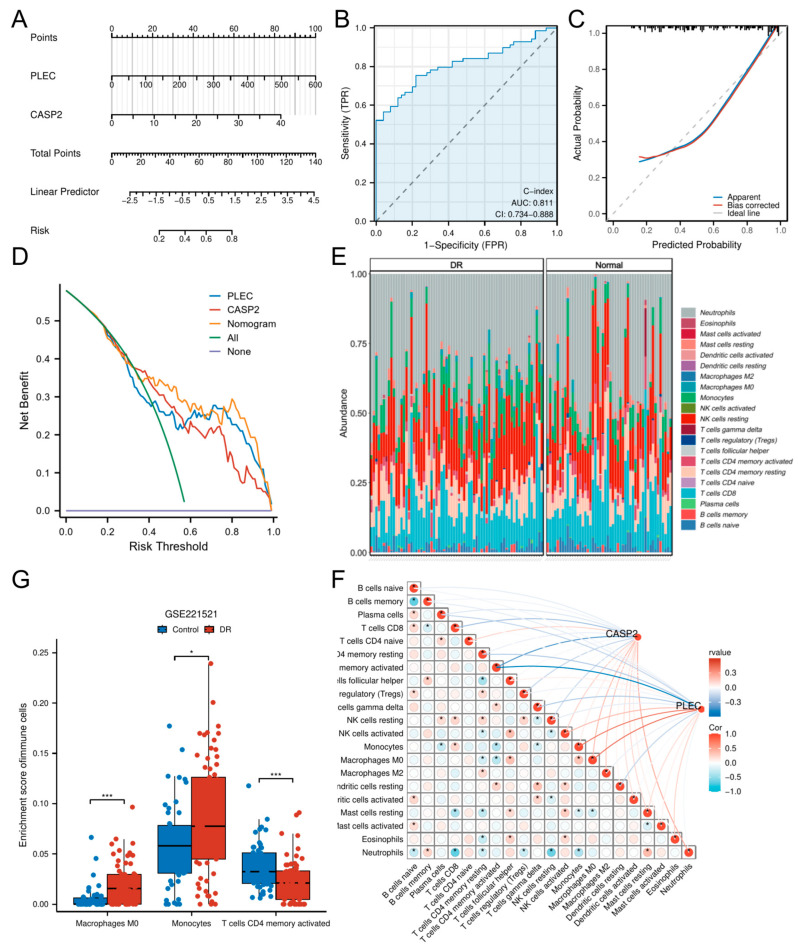
Construction of a diagnostic nomogram and immune infiltration analysis. (**A**) A predictive nomogram incorporating the hub genes CASP2 and PLEC for assessing DR risk. (**B**) ROC curve analysis evaluating the discriminatory ability of the nomogram. (**C**) Calibration plot evaluating the agreement between predicted and observed outcomes. (**D**) DCA showing the clinical utility of the nomogram across various risk thresholds. (**E**) Relative abundances of 21 immune cell types between DR and control cohorts. (**F**) Box plots showing immune cell types with significantly different infiltration levels between DR and control cohorts. (**G**) Correlation matrix of immune cell types and their associations with CASP2 and PLEC expression. (* *p* < 0.05; *** *p* < 0.001).

A hub gene-based nomogram was constructed for DR risk prediction (Figure 5A). The model exhibited favorable discriminative ability with a concordance index (C-index) of 0.811 (95% CI: 0.734–0.888; Figure 5B). Calibration curves demonstrated no significant deviation between predicted and observed outcomes (goodness-of-fit *p* = 0.0515 > 0.05; Figure 5C), indicating optimal model fit. DCA further established the superior net clinical benefit of the nomogram compared to single predictors across threshold probabilities (Figure 5D).

### 3.5. Immune Microenvironment Characterization

To delineate the immune landscape in DR, we employed CIBERSORT to quantify 21 immune cell subsets. Significant alterations were observed in DR patients versus controls (Figure 5E), with marked enrichment of Macrophages M0 (*p* < 0.001) and Monocytes (*p* < 0.05), concomitant with reduced activated CD4+ memory T cells (*p* < 0.001) (Figure 5F).

Correlation analyses revealed CASP2 expression negatively correlated with CD8+ T cells (r = −0.258, *p* < 0.01) and activated CD4+ memory T cells (r = −0.432, *p* < 0.001), while showing positive correlations with Macrophages M0 (r = 0.209, *p* < 0.05) and Neutrophils (r = 0.261, *p* < 0.01). PLEC expression negatively associated with resting CD4+ memory T cells (r = −0.222, *p* < 0.05), gamma delta T cells (r = −0.223, *p* < 0.05), and activated CD4+ memory T cells (r = −0.593, *p* < 0.001), but positively correlated with Monocytes (r = 0.417, *p* < 0.001), Macrophages M0 (r = 0.441, *p* < 0.001), and activated NK cells (r = 0.215, *p* < 0.05) (Table S8). Comprehensive correlation matrices are visualized in Figure 5G.

### 3.6. GSEA of Hub Genes and ceRNA Network

**Figure 6:**
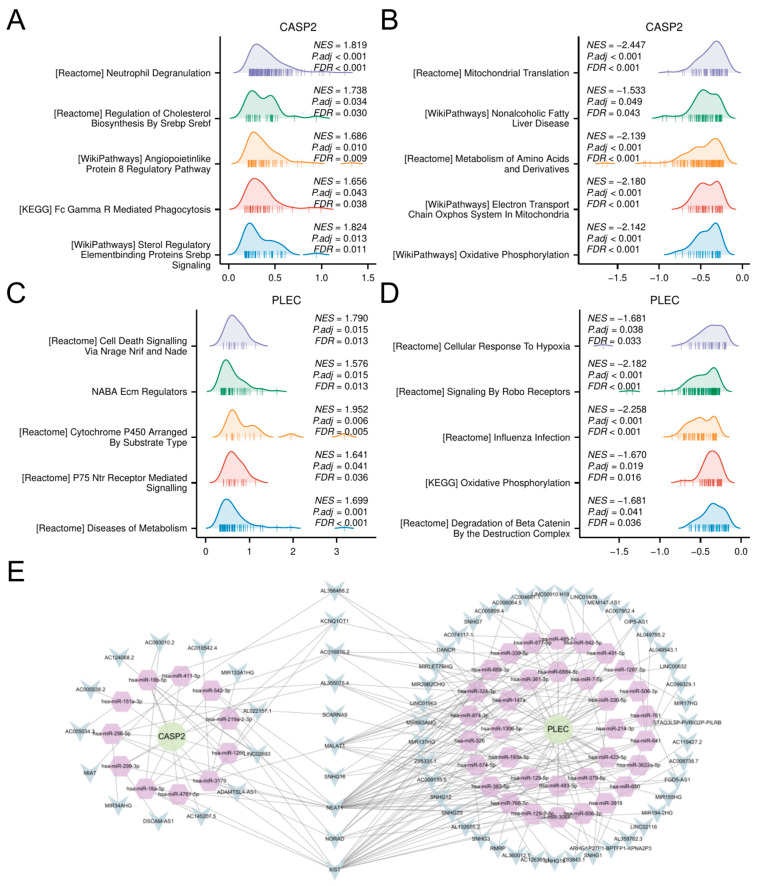
GSEA and ceRNA network construction. (**A**,**B**) GSEA results for samples stratified by high (**A**) and low (**B**) expression of CASP2. (**C**,**D**) GSEA results for samples stratified by high (**C**) and low (**D**) expression of PLEC. (**E**) The constructed ceRNA regulatory network. Circles: hub genes (CASP2, PLEC); Hexagons: miRNAs; V-shapes: lncRNAs.

Samples were stratified by median expression levels of CASP2 and PLEC for differential analysis. GSEA delineated distinct functional themes associated with hub gene expression dynamics. High CASP2 expression primarily drove innate immune activation, exemplified by neutrophil degranulation and Fc Gamma R Mediated Phagocytosis (*p* < 0.001), coupled with lipid metabolic reprogramming via SREBP signaling (Figure 6A). Conversely, low CASP2 levels activated mitochondrial energy metabolism pathways, including oxidative phosphorylation and electron transport chain activity (Figure 6B). For PLEC, high expression promoted cell death mechanisms (e.g., NRAGE/NRIF/NADE signaling) and extracellular matrix dysregulation (Figure 6C), whereas low expression triggered hypoxia adaptation (ROBO signaling) and metabolic stress responses (Figure 6D).

Furthermore, to investigate the potential post-transcriptional regulation of CASP2 and PLEC, a ceRNA network was constructed. Using the ENCORI platform, we identified experimentally supported interactions (≥5 supporting CLIP-seq datasets), resulting in a potential regulatory network comprising the two candidate genes, 45 miRNAs, and 64 lncRNAs (Figure 6E; complete interaction details in Table S9).

### 3.7. scRNA-Seq Analysis

**Figure 7:**
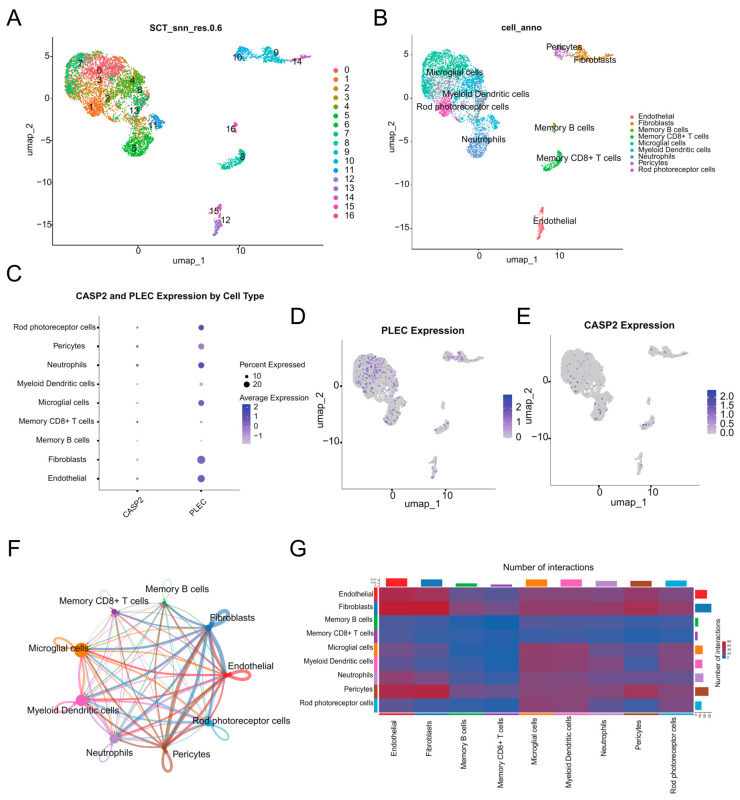
scRNA-seq analysis. (**A**) UMAP visualization of the 17 distinct cell clusters identified through unsupervised clustering. (**B**) UMAP plot annotated with major cell types. (**C**) Dot plot visualizing the expression level and percentage of expressing cells for CASP2 and PLEC across all cell types. (**D**) UMAP feature plot showing the expression distribution of PLEC. (**E**) UMAP feature plot showing the expression distribution of CASP2. (**F**) Circle plot of cell–cell communication, where the edge width represents the interaction strength between cell types. (**G**) Heatmap depicting the number of significant ligand-receptor interactions between each cell type pair.

scRNA-seq analysis of five DR FVM samples identified 6745 high-quality cells, delineating a complex ecosystem composed of immune and vascular-stromal compartments (Figure 7A,B). The immune compartment was dominated by microglial cells and included infiltrating myeloid dendritic cells, neutrophils, memory CD8+ T cells, and memory B cells. The vascular-stromal compartment, central to pathological neovascularization and fibrosis, consisted of endothelial cells, pericytes, and fibroblasts. A minor population of rod photoreceptor cells was also detected, likely representing entrapped retinal neurons.

Interrogation of hub genes revealed distinct expression patterns (Figure 7C–E). PLEC exhibited broad and robust expression across major cell types, with high mean expression (>0.20) and substantial expression percentages in Neutrophils, Endothelial cells, Rod photoreceptor cells, Fibroblasts, and Microglial cells (Figure 7D). CASP2 showed its highest mean expression level in Memory CD8+ T cells, with significant expression also detected in Neutrophils and Pericytes (Figure 7E).

Cell–cell communication analysis revealed extensive interaction networks within the DR microenvironment (Figure 7F,G). Fibroblasts and Endothelial cells emerged as communication hubs, demonstrating the highest number of outgoing and incoming interactions. Microglial cells showed prominent signaling to neuronal elements and other immune populations. Neutrophils and Pericytes also participated actively in the communication network, while adaptive immune cells showed minimal involvement in cell–cell signaling.

### 3.8. Validation of Hub Gene Expression

**Figure 8:**
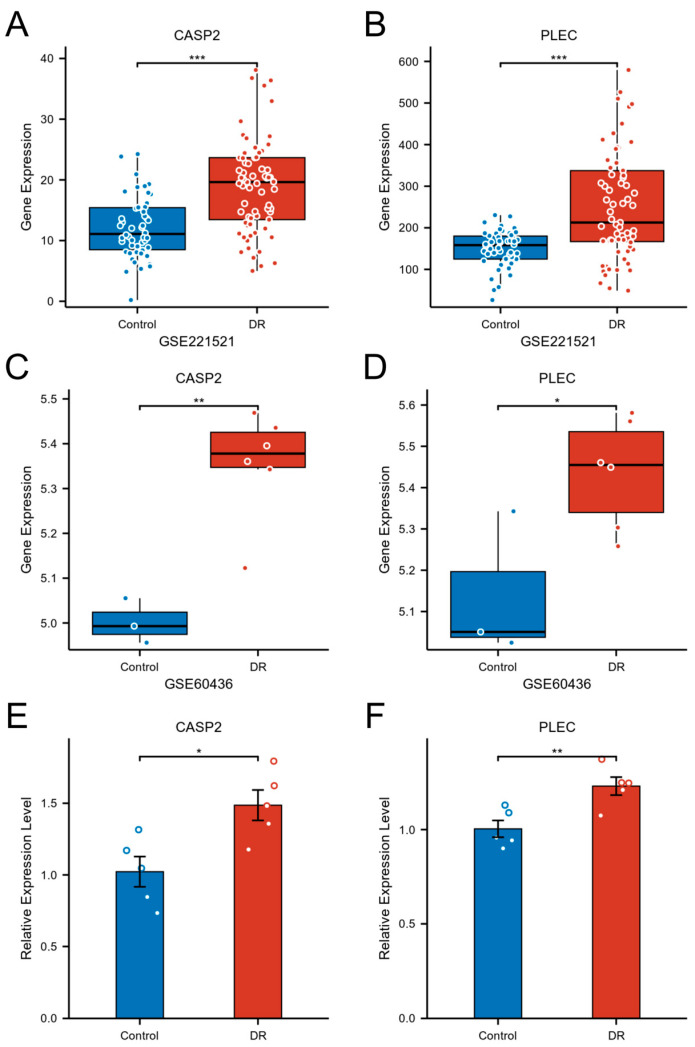
Validation of CASP2 and PLEC expression in DR. (**A**,**B**) Expression levels of CASP2 (**A**) and PLEC (**B**) were significantly upregulated in the blood of DR patients (GSE221521). (**C**,**D**) Expression levels of CASP2 (**C**) and PLEC (**D**) were significantly upregulated in FVM from DR patients (GSE60436). (**E**,**F**) Relative mRNA expression levels of CASP2 (**E**) and PLEC (**F**), measured by RT-qPCR, were significantly increased in hRMECs cultured under HG conditions compared to NG controls. (* *p* < 0.05; ** *p* < 0.01; *** *p* < 0.001).

The expression and pathological relevance of the hub genes CASP2 and PLEC were rigorously validated through a multi-tiered approach. Initial analysis in the peripheral blood discovery cohort (GSE221521) confirmed their significant upregulation in DR, with CASP2 exhibiting a log2FC of 0.598 (adjusted *p*-value < 0.001) and PLEC a log2FC of 0.702 (adjusted *p*-value < 0.001) (Figure 8A,B). This finding was further substantiated in an independent FVM dataset (GSE60436), where both genes were significantly elevated, with CASP2 at log2FC = 0.352 (adjusted *p*-value = 0.014) and PLEC at log2FC = 0.296 (adjusted *p*-value = 0.040) (Figure 8C,D). Finally, expression validation in a HG cell model of DR demonstrated a 1.49-fold increase in CASP2 expression (*p* = 0.014) and a 1.23-fold increase in PLEC expression (*p* = 0.008) compared to normal glucose controls (Figure 8E,F). This consistent evidence across multiple levels—from peripheral blood to primary retinal tissue and a disease-relevant cellular context—firmly establishes CASP2 and PLEC as robustly validated candidates intimately associated with DR.

## 4. Discussion

Leveraging an integrative analysis of multi-omics data, this study provides the first systematic investigation into the potential role of oxeiptosis—an ROS induced, caspase-independent cell death pathway—in the pathogenesis of DR. By harmonizing transcriptomic profiling from peripheral blood, putative causal inference via MR, machine-learning feature selection, and multi-tiered validation including single-cell transcriptomics of fibrovascular membranes, we nominate CASP2 and PLEC as key candidates mechanistically linked to oxeiptosis-related pathways, with implications for immune dysregulation, cytoskeletal remodeling, and clinical stratification in DR [6,7].

Our analytical pipeline first identified 196 DE-ORGs that were differentially expressed in DR and co-expressed within modules strongly correlated with the oxeiptosis phenotype. Functional enrichment analysis revealed their significant convergence on established DR pathways, including apoptosis regulation, IL-17-mediated inflammatory signaling, and calcium ion homeostasis [44,45], thereby linking oxeiptosis-associated genes to core DR pathology. To elevate these associations beyond mere correlation, we employed Mendelian randomization (MR) analysis, which provided putative causal evidence for five of these DE-ORGs. Among them, CASP2 and PLEC were subsequently validated as independent risk factors through complementary machine learning approaches and multivariable regression, solidifying their status as the most robust candidate hub genes.

CASP2 emerged as a compelling, though mechanistically nuanced, candidate. Its significant upregulation across DR blood samples, fibrovascular membranes, and high glucose-stressed hRMECs aligns with its established role as a sensor of oxidative stress [46]. While oxeiptosis is defined as caspase-independent in its execution, our data suggest that CASP2 may function not as an executor, but as a critical upstream regulator or a parallel stress-sensing pathway that influences the cellular response to oxidative damage in the diabetic milieu. This hypothesis is supported by GSEA, which linked high CASP2 expression to the activation of potent innate immune pathways such as neutrophil degranulation and FcγR-mediated phagocytosis [47,48,49]. Most intriguingly, our single-cell analysis revealed a strikingly specific enrichment of CASP2 in memory CD8+ T cells within the DR microenvironment. This novel finding, coupled with its significant negative correlation with T cell abundances in peripheral blood, suggests a previously unexplored role for CASP2 in regulating T-cell activation, survival, or cytokine production, directly contributing to the pro-inflammatory shift in DR. Thus, CASP2 appears to be a nexus linking oxidative stress to immune dysregulation, warranting dedicated future investigation.

Conversely, PLEC appears to be a central mediator of the structural pathology in DR. As a giant cytoskeletal scaffold protein that serves as a “molecular bridge” [50], its marked overexpression in fibroblasts, endothelial cells, and microglial cells—key actors in fibrovascular membrane formation [22]—positions it as a master regulator of tissue architecture and an active driver of pathological remodeling. We hypothesize that PLEC overexpression reinforces cellular rigidity, disrupts barrier function, and facilitates aberrant cell migration, thereby providing a scaffold for excessive ECM deposition. This is consistent with our GSEA linking high PLEC expression to pathways like NRAGE-induced cell death and collagen organization [51,52]. Furthermore, as a signaling platform, PLEC may integrate oxidative and inflammatory signals to drive pro-fibrotic gene expression (e.g., collagen production), thereby directly translating the diabetic milieu into the structural pathology that characterizes advanced DR.

Collectively, our data support a synergistic model of DR pathogenesis wherein CASP2-mediated immune dysregulation and PLEC-driven structural remodeling engage in a self-reinforcing cycle. Inflammatory signals potentiated by CASP2 can activate fibroblasts and endothelial cells, thereby increasing PLEC expression and pathological matrix deposition. Conversely, a stiff, remodeled extracellular matrix can further promote inflammation and oxidative stress, perpetuating the disease process. This model nominates both hub genes as potential nodes for therapeutic intervention aimed at breaking this cycle.

The remaining causal genes provide additional mechanistic insights: CYP27A1 encodes cholesterol 27-hydroxylase, and its deficiency has been associated with retinal oxidative stress and vascular changes resembling diabetic retinopathy in experimental models [53]; GPD2 encodes a key enzyme in the glycerol-3-phosphate shuttle, with evidence showing its protective role against diabetic complications through mitochondrial metabolism regulation [54]; and FBN2 encodes an extracellular matrix protein, though its specific role in diabetic retinopathy requires further validation [55]. These associations further underscore the involvement of metabolic dysregulation and matrix remodeling in DR pathogenesis.

Our immune infiltration analysis revealed profound microenvironment shifts in DR, including expansion of M0 macrophages and monocytes, alongside altered activated CD4+ memory T cell populations. The correlation of both hub genes with M0 macrophages—plastic precursors that can polarize toward pro-angiogenic M2 phenotypes—may contribute to sustaining retinal inflammation and pathological neovascularization [56]. This immune landscape parallels findings in other diabetic complications, suggesting shared pathogenic mechanisms amenable to immunomodulatory interventions [57,58].

Our integrated analyses suggest a potential translational pathway for CASP2 and PLEC, highlighting their dual promise as biomarkers for early detection and as candidates for therapeutic development. The robust performance of our blood-based nomogram (C-index = 0.811) indicates the feasibility of developing a minimally invasive liquid biopsy assay. Such a test could improve current DR screening paradigms by enabling the stratification of diabetic patients based on molecular risk profiles, thus facilitating earlier intervention. From a therapeutic perspective, the putative causal roles of CASP2 and PLEC nominate them as promising intervention targets. We hypothesize that inhibiting CASP2 might dampen chronic inflammatory responses, while modulating PLEC function could impede pathological fibrovascular remodeling. The identified ceRNA network further suggests upstream regulatory nodes that could be explored for RNA-based therapeutic strategies. These insights position CASP2 and PLEC as molecules of considerable interest for future translational research in DR.

This study has several limitations that should be considered when interpreting the results. First, our putative causal inferences from MR rely on blood-derived cis-eQTLs from the GTEx consortium, which comprises predominantly European ancestry individuals [59]. This may limit the generalizability of our findings to other populations, and future studies using multi-ethnic and, ideally, retina-specific eQTLs are warranted. Second, the sample sizes of our validation cohorts were limited, particularly the FVM dataset, and the scRNA-seq data were derived exclusively from proliferative DR patients, highlighting the need for validation across the full spectrum of DR severity. Most importantly, as a bioinformatics study, our work is inherently hypothesis-generating. The transcriptomic-centric nature of our analysis necessitates future protein-level validation and, crucially, functional experiments to definitively establish the mechanistic roles of CASP2 and PLEC in oxeiptosis and DR pathogenesis. Future work should therefore prioritize functional validation in diabetic models and the exploration of the identified ceRNA regulators.

## 5. Conclusions

In summary, this study provides the first multi-omics evidence linking the oxeiptosis pathway to DR pathogenesis. We identify CASP2 and PLEC as putatively causal hub genes, delineate their association with distinct pathological processes (immune dysregulation and fibrotic remodeling), and propose testable hypotheses for their functional roles. This work paves the way for subsequent functional studies and translational development aimed at the early detection and intervention of DR.

## Data Availability

The transcriptomic datasets (GSE221521, GSE60436, and GSE165784) analyzed during this study were downloaded from the Gene Expression Omnibus (GEO) repository (https://www.ncbi.nlm.nih.gov/geo/, 1 August 2025). The oxeiptosis-related genes were identified from the GeneCards database (https://www.genecards.org/, 1 August 2025). The blood cis-eQTL data were acquired from the GTEx Portal (https://gtexportal.org/home/, version 8, 1 August 2025). The genome-wide association study (GWAS) summary statistics for diabetic retinopathy were obtained from the FinnGen R12 database (https://r12.finngen.fi/, 1 August 2025). The microRNA interactions were sourced from the ENCORI platform (https://rnasysu.com/encori/, 3 August 2025). All data supporting the findings of this study are publicly available from the aforementioned databases. All datasets generated and analyzed during this research are included in this published article and its Supplementary Information Files. Correspondence and requests for materials should be addressed to the corresponding authors.

## Supplementary Materials

The following supporting information can be downloaded at https://www.mdpi.com/article/10.3390/biomedicines13112789/s1, Figure S1: Scatter plots of the exposure-outcome; Figure S2: Forest plots of the effect sizes; Figure S3: Funnel plots assessing the symmetry of instrumental variables for the MR analyses; Figure S4: Leave-one-out sensitivity analysis; Table S1: List of oxeiptosis-related genes (ORGs) used in this study; Table S2: Marker genes used for automated cell-type annotation with ScType; Table S3: The list of 196 candidate DE-ORGs; Table S4: GO and KEGG enrichment results (DE-ORGs); Table S5: Results of heterogeneity tests; Table S6: Results of the MR-Egger intercept test; Table S7: Results of the MR-PRESSO global test; Table S8: Correlation between the expression of hub genes and immune cell infiltration levels. Table S9: ceRNA network interaction details.

## Abbreviations

The following abbreviations are used in this manuscript:

DR	Diabetic retinopathy
MR	Mendelian randomization
ROS	Reactive oxygen species
GEO	Gene Expression Omnibus
FVM	Fibrovascular membrane
scRNA-seq	Single-cell RNA sequencing
ORGs	Oxeiptosis-related genes
cis-eQTL	Cis-expression quantitative trait loci
GTEx	Genotype-Tissue Expression
GWAS	Genome-wide association study
SNPs	Single-nucleotide polymorphisms
DEGs	Differentially expressed genes
ssGSEA	Single-sample gene set enrichment analysis
WGCNA	Weighted gene co-expression network analysis
GO	Gene Ontology
KEGG	Kyoto Encyclopedia of Genes and Genomes
PPI	Protein–protein interaction
ORs	Odds ratios
ROC	Receiver operating characteristic
AUC	Area under the curve
IVW	Inverse-variance weighted
SVM-RFE	Support vector machine recursive feature elimination
CIs	Confidence intervals
DCA	Decision curve analysis
GSEA	Gene Set Enrichment Analysis
FDR	False discovery rate
lncRNA	Long non-coding RNA
miRNA	microRNA
TOM	Topological overlap matrix
