# Supplementary figures and images for: An Integrated Multi-Omics Analysis Identifies Oxeiptosis-Related Biomarkers in Diabetic Retinopathy
Source: Biomedicines. 2025 Nov 15;13(11):2789. doi: 10.3390/biomedicines13112789 (PMC12650381; doi:10.3390/biomedicines13112789)

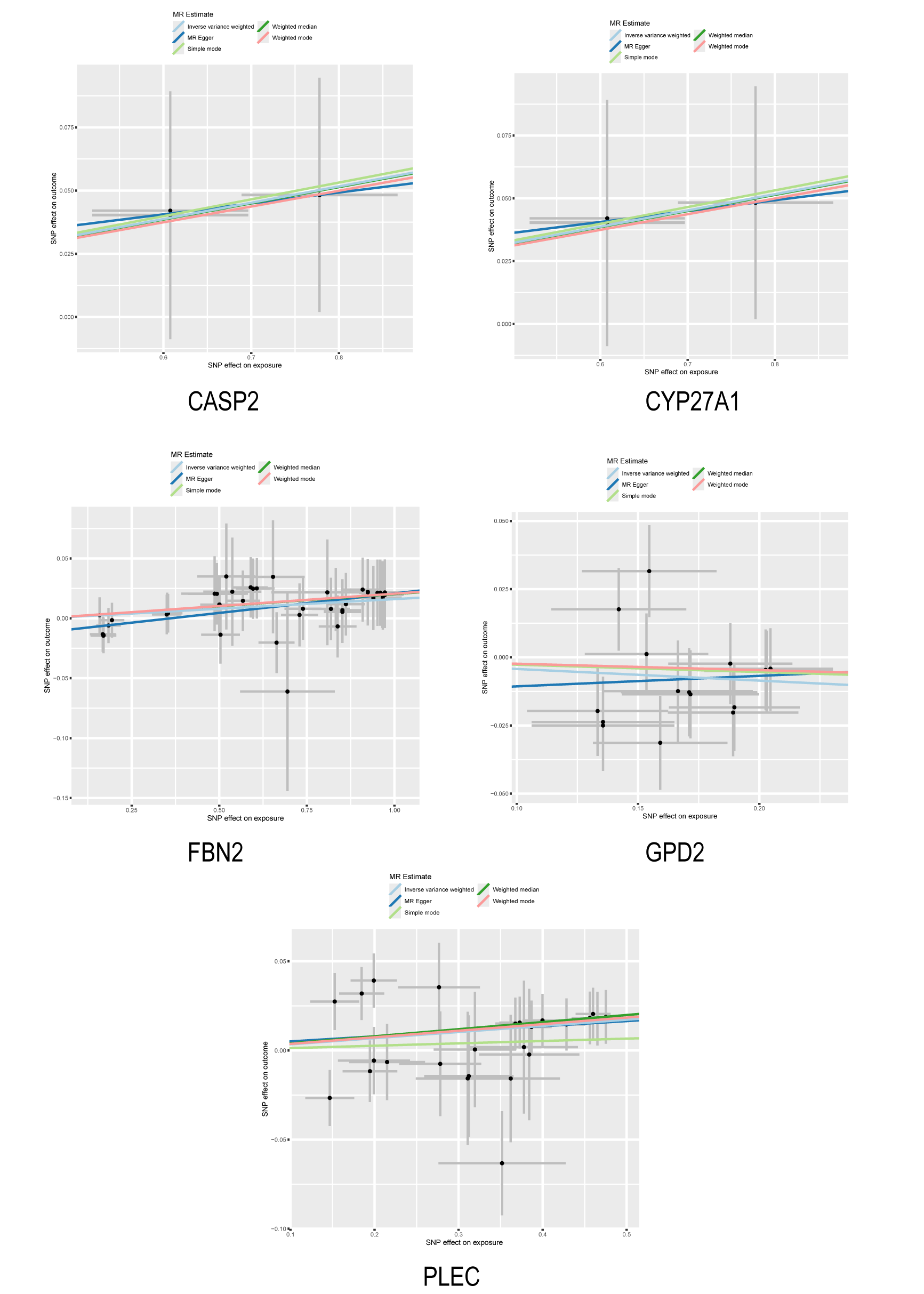

Supplement: Supplementary file 1 [file biomedicines-13-02789-s001.zip › Figure S1.tif]

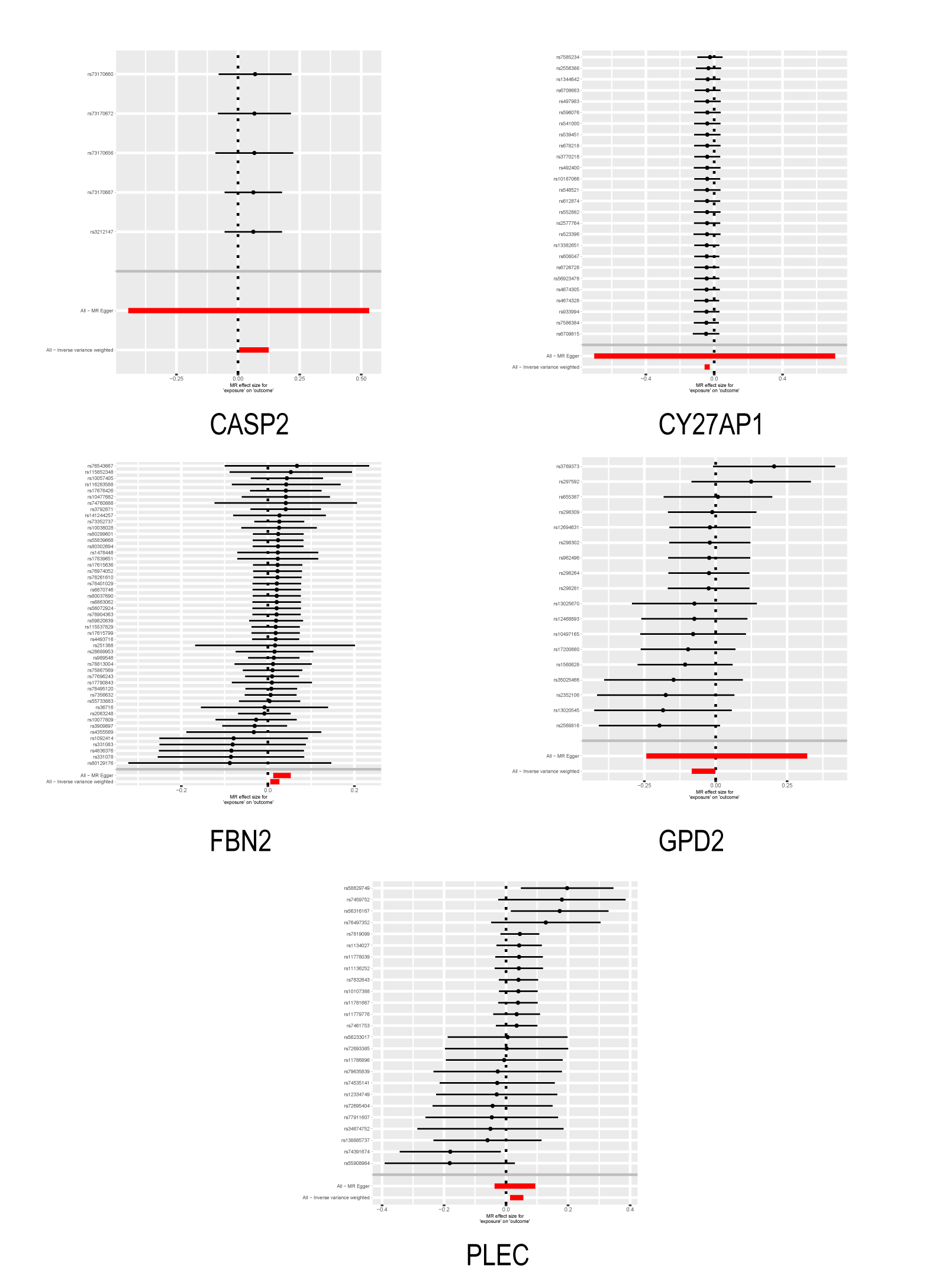

Supplement: Supplementary file 1 [file biomedicines-13-02789-s001.zip › Figure S2.tif]

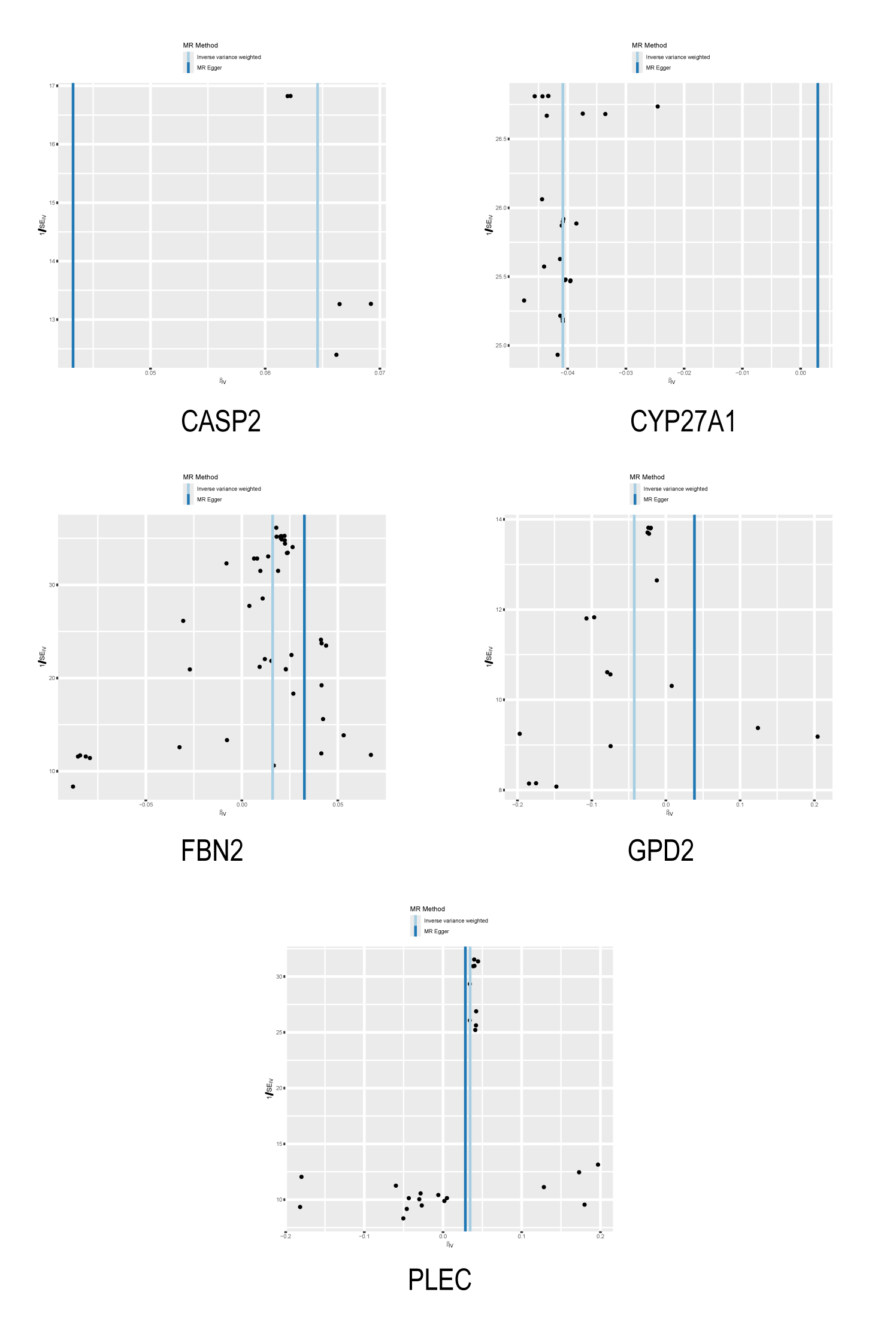

Supplement: Supplementary file 1 [file biomedicines-13-02789-s001.zip › Figure S3.tif]

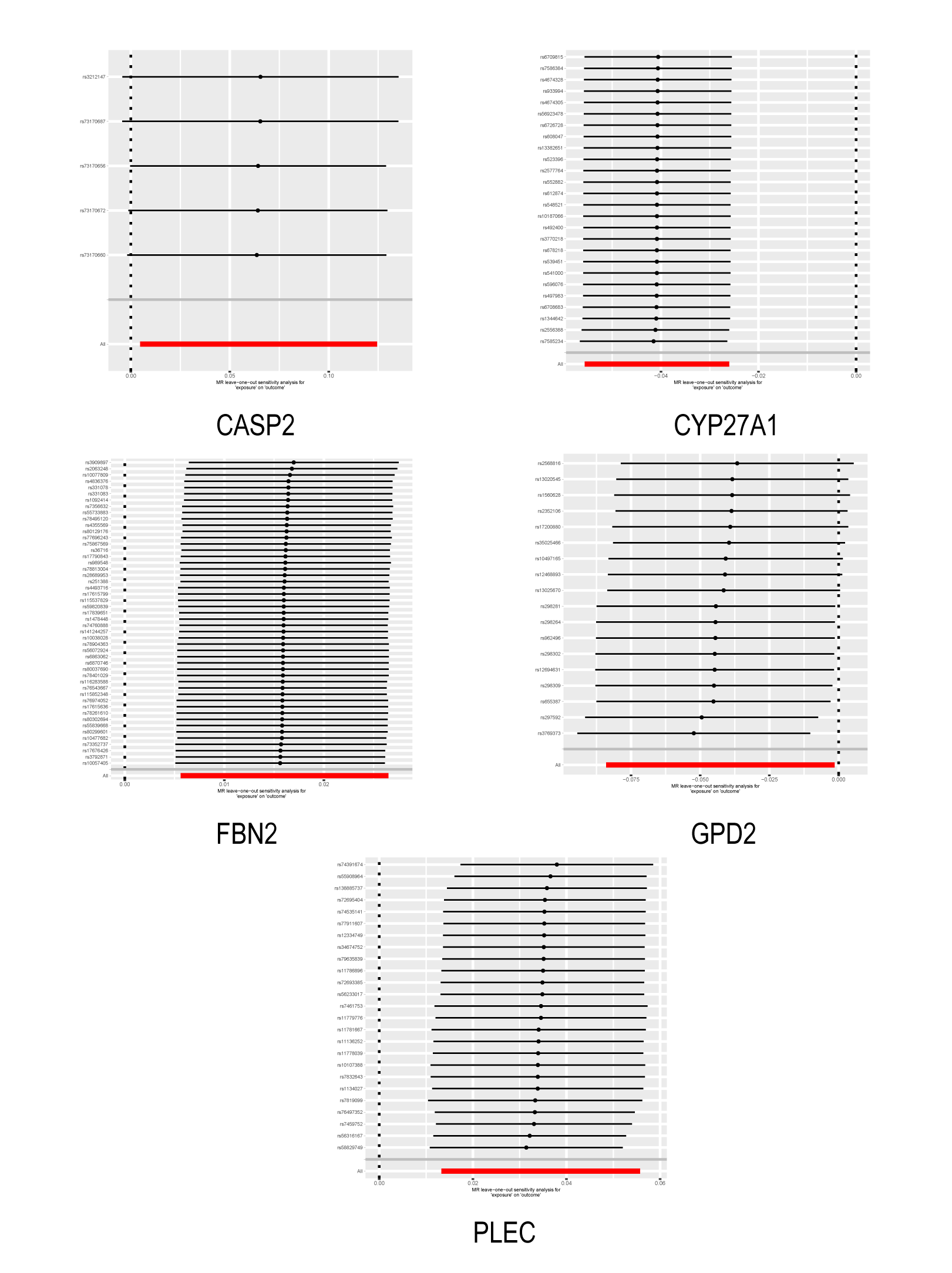

Supplement: Supplementary file 1 [file biomedicines-13-02789-s001.zip › Figure S4.tif]
